# Effects of Lee Silverman Voice Treatment-BIG on Motor, Cognition, Mental Health, Occupational Performance, and Occupational Balance in Patients With Schizophrenia: A Single-Subject Experimental Study

**DOI:** 10.1155/oti/3566653

**Published:** 2025-02-19

**Authors:** Woo-Hyuk Jang, Sang-Min Seo, Si-Hyun Kim

**Affiliations:** ^1^Department of Occupational Therapy, College of Health Science, Kangwon National University, Samcheok-si, Republic of Korea; ^2^Department of Occupational Therapy, College of Health & Biotechnology, Semyung University, Jecheon-si, Republic of Korea; ^3^DA & You Specialized Rehabilitation Exercise Center, Seoul, Republic of Korea

## Abstract

**Introduction:** In this study, we aim to apply BIG to patients with schizophrenia to confirm changes in mental symptoms, task performance, and occupational balance through motor and cognitive enhancement.

**Method:** This study used a single-subject A-B-A design. It consisted of a total of 22 sessions, with 4 sessions in the baseline period, 16 sessions in the intervention period (Lee Silverman Voice Treatment-BIG), and 2 sessions in the follow-up period. The subjects were three male patients diagnosed with chronic schizophrenia, all severe cases. During the 22 sessions, the timed up and go test (TUG) and functional reach test (FRT) and the Montreal Cognitive Assessment (MoCA) were used to determine changes in motor function and cognition, and the subjective cognitive and mental score (SS), Canadian Occupational Performance Measure (COPM), and Occupational Balance Questionnaire-Korean (OBQ-K) were used to determine changes in psychiatric symptoms, work performance, and work balance satisfaction before and after the intervention. Statistically significant changes were determined using the two standard deviation (2SD) band method.

**Results:** The TUG, FRT, and MoCA showed significant results in the intervention period compared to the baseline period. The SS, COPM, and OBQ-K also showed positive changes in scores from pre- to postintervention.

**Conclusion:** In this study, BIG was found to promote improvement in motor and cognitive function in chronic schizophrenia patients, with positive effects on psychiatric symptoms, task performance, and occupational balance satisfaction.

## 1. Introduction

Schizophrenia is a mental disorder that can lead to a loss of social functioning through distorted language and behavior, showing both positive symptoms of excessive normal functioning and negative symptoms due to reduction [1, 2]. The positive symptoms mainly include hallucinations, delusions, and disorganized behavior, while the negative symptoms include a decrease or absence of functions such as movement, thinking, emotions, and motivation [1, 2]. Schizophrenia is a common disorder affecting approximately 1 in 100 people worldwide and typically begins in the late teens to early 30s [3, 4]. The nature of the disease is one of long duration, with the possibility of recovery after onset, but a tendency toward chronicity due to repeated relapses, requiring lifelong management rather than a complete cure [5]. The primary treatment for schizophrenia is medication, which must be taken regularly every day, with effective results for positive symptoms but limited efficacy for negative symptoms [6, 7].

Another issue is that schizophrenia is often reported to be associated with serious neurological and metabolic side effects [8, 9]. In addition, the risk of early death due to cardiovascular disease is reported to be two to three times higher than in the general population [9], with obesity reported to play a significant role in these triggering factors [10]. In addition, excessive consumption of high-fat, high-sugar foods to alleviate psychological problems such as stress and depression has been reported to exacerbate these risks [11, 12]. As a result, the importance of managing symptoms and complications in patients with schizophrenia has been emphasized, but clear interventions have not yet been developed [13]. Furthermore, it is predicted that the number of chronic schizophrenia patients will continue to increase, as of 2022, in the era of global aging, when 22 countries will enter the super-elderly society [14–16].

On the other hand, one of the side effects of treating schizophrenia with antipsychotic medications is the potential development of drug-induced parkinsonism if the medication is taken continuously [17]. While the main mechanism of onset in schizophrenia is hyperactivity of the mesolimbic dopaminergic pathway associated with the ventral tegmental area [18], Parkinson's disease (PD) is primarily characterized by symptoms such as bradykinesia due to decreased dopamine secretion in the nigrostriatal pathway [19]. Despite the differences in the neural pathways and mechanisms that cause the two conditions, the side effects of treating schizophrenia can increase the risk of developing Parkinson's symptoms later in life [20]. In addition, both conditions lead to a decrease in quality of life due to common psychiatric symptoms such as hallucinations and delusions, as well as various cognitive and motor impairments [17, 19, 21].

In this way, various nonpharmacological interventions have been reviewed to address various issues seen in patients with schizophrenia [22–25]. First, a review of the effects of exercise intervention in patients with schizophrenia was conducted, focusing on 20 studies [22]. This study reported that although exercise intervention did not have a significant effect on body mass index in patients with schizophrenia, it did have effects on physical fitness and cardiometabolic risk factors. It also reported that moderate-to-vigorous exercise for approximately 90 min per week was effective in improving psychiatric symptoms, motor function, and cognitive function. The next study, a review of the effects of aerobic exercise interventions on clinical symptoms, quality of life, overall functioning, depression, and cognition was conducted in patients with schizophrenia through 29 experimental studies (involving 1109 individuals) [23]. According to the research results, aerobic exercise had significant effects in all areas, including improvement in clinical symptoms. Yoga was also reported to be an effective exercise for improving long-term memory. Another study reviewed the effects of aerobic exercise and cognitive improvement in patients with schizophrenia through 10 experimental studies (involving 385 individuals) and 15 experiments (involving 593 individuals) [24, 25]. The results of the two review studies showed significant improvements in global cognition, attention/vigilance, working memory, and language learning with aerobic exercise. In addition, higher doses of exercise supervised by exercise professionals were found to be more effective, and significant improvements in global cognition were observed when group exercise and exercise professional supervision were provided for more than 90 min per week and for more than 12 weeks [25]. Thus, research on various exercise programs as nonpharmacological interventions and their effects has been demonstrated.

On the other hand, Lee Silverman Voice Treatment (LSVT)-BIG Exercise (hereafter referred to as BIG) has recently attracted attention [26, 27]. This LSVT was developed in 1987 for patients with PD [28, 29] and is divided into LOUD for speech intervention and BIG for motor function intervention [30]. The highly effective composition of BIG for motor function consists of a total of four4 components as follows: maximal daily exercise, which consists of seven basic exercises performed daily; functional component tasks, which consists of five simple daily tasks without grading; hierarchy tasks, which consists of complex tasks used in real daily life in a sequential manner; and BIG walking, which involves walking with large steps [30]. BIG has been mainly used as a treatment to overcome bradykinesia and hypokinesia in PD patients among motor functions [31]. BIG is a high-intensity exercise designed to produce larger and stronger proprioceptive sensations when performing individual movements [32]. This requires approximately 60 min per session, with training performed four consecutive days per week for 4 weeks, and the duration of home program performance on training days (once a day, 15–20 min) is different from nontraining days (twice a day, 15–20 min each) as part of the protocol [30]. Numerous studies have reported on the effects of BIG [33–39]. Results from the implementation of BIG in Parkinson's patients showed improvements in motor function [33–35, 40] and a decrease in the depression index [36, 37]. In addition, research on various diseases other than PD has also been reported [38, 39]. After BIG, the effects of improving motor function and increasing job satisfaction in stroke patients [38] and improving balance and mobility speed in idiopathic normal pressure hydrocephalus patients [39] have been reported. The effects of BIG have been reported not only on motor function but also on satisfaction, depression, and other psychological domains in various diseases. However, research on patients with schizophrenia is still lacking. Therefore, in this study, we hypothesized that BIG would be effective in improving various symptoms, including motor function, in patients with schizophrenia.

In this study, we aim to apply BIG to patients with schizophrenia to confirm changes in mental symptoms, task performance, and occupational balance through motor and cognitive enhancement. Additionally, we seek to provide evidence for a new intervention technique to improve various symptoms associated with disorders such as schizophrenia.

## 2. Method

### 2.1. Study Design and Subjects

This study used a single-subject A-B-A design. It consisted of a total of 22 sessions, with 4 sessions in the baseline period, 16 sessions in the intervention period, and 2 sessions in the follow-up period. The subjects were three male patients diagnosed with chronic schizophrenia, all severe cases. They are currently living in a group home in a psychiatric rehabilitation facility and are employed part time in a job arranged by the facility. All subjects are smokers and do not consume alcohol. The inclusion criteria were based on the minimum motor function to perform BIG and the characteristics of people with schizophrenia, as follows: (1) individuals diagnosed with schizophrenia for more than 10 years and currently living in the local community, (2) individuals without cardiovascular disease who can walk independently and perform aerobic exercise, and (3) individuals with a Mini-Mental State Examination (MMSE) score of 24 or higher. The general characteristics of the subjects are as follows (Table 1).

### 2.2. Study Process

**Figure 1:**
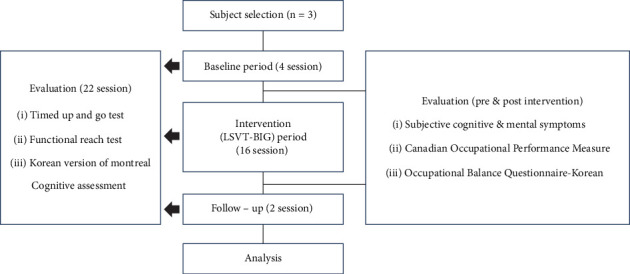
Study processing diagram. Abbreviation: LSVT: Lee Silverman Voice Treatment.

In this study, interventions were conducted with subjects A, B, and C in sequence from August 2023 to December 2023. An occupational therapist with experience in psychiatric institutions and completion of the BIG training course conducted the interventions by visiting the group home directly and by conducting the interventions in a separate, independent room on the same day and time each week. Physical and cognitive functions were assessed after 22 sessions, while psychiatric symptoms, task performance, and occupational balance were assessed before and after the interventions (Figure 1). Prior to the experiment, the purpose of the research was explained to the subjects and their legal guardians, and the research was conducted only with those who gave written consent after sufficient understanding. This study was conducted following the deliberation of the Kangwon National University Institutional Review Board (IRB) (KWNUIRB-2023-08-002-001).

### 2.3. Intervention Methods

The BIG program was performed for one session, 60 min per session, four sessions per week, for a total of 4 weeks. The BIG program was delivered according to standardized protocols (Table 2). The occupational therapist videotaped the BIG process and provided education to facility staff, enabling them to perform Home Work. Due to the nature of the group home, there was a staff member on duty every day, even on weekends to monitor the continuation of BIG. During BIG, maximal daily exercise was uniformly applied to three participants according to predetermined movements, while functional component tasks and hierarchy tasks had to be tailored and applied to the participants' needs and abilities (Table 3). Functional component tasks and hierarchy tasks were individually designed for each participant after identifying/analyzing the behaviors that require intervention in daily life in the group home. In addition, BIG walking training was conducted with various adjustments in time, distance, and surfaces to allow participants to perform significant walking indoors, on rooftops, outdoors, or in other major living environments. Subsequently, in Home Work, ongoing training was integrated into daily life to repeat and establish habits by linking individual roles (cleaning, cooking, hobbies, etc.) in a group home setting.

### 2.4. Evaluation Tools

#### 2.4.1. Timed Up and Go Test (TUG)

The TUG was used to assess dynamic balance and walking ability. The measurement procedure involves sitting in a chair with armrests at a height of 50 cm, rising from the chair at the start signal of the experimenter, returning to a point 3 m away, and measuring the time taken to return to the chair. This test has an intrarater reliability of *r* = 0.99 and interrater reliability of *r* = 0.98, and in terms of score interpretation, a lower time in seconds indicates better performance [41].

#### 2.4.2. Functional Reach Test (FRT)

The FRT was used to test the dynamic balance ability, which is measured by extending the arms forward in parallel with the elbows extended and the shoulders flexed at about 90°, keeping one arm horizontal, and leaning forward at the waist as far as possible. The change in distance (centimeter) from the starting position to the final position is measured at the third metacarpophalangeal joint. The test–retest reliability is *r* = 0.92, and the interrater reliability is *r* = 0.98, and for score interpretation, the higher the change in centimeter, the better [42].

#### 2.4.3. Korean Version of Montreal Cognitive Assessment (MoCA)

The MoCA was used to assess cognitive function. This tool is designed to screen patients with mild cognitive impairment and consists of visuospatial executive function (5 items), vocabulary (3 items), attention (8 items), sentence production (3 items), abstractions (2 items), delayed recall (5 items), and orientation (6 items) [43]. In this study, we used the Korean version of the MoCA, which was adapted into Korean, and the measurement method is a total of 30 out of 30 points, with 1 point added for subjects with 6 years of education or less, and the assessment is administered within approximately 10–15 min. The MoCA has a reliability of 0.86, which means that a score of 23 or higher is considered normal, but a score of 22 or lower is a screening test for mild cognitive impairment. Higher scores are also better [44]. Permission to use MoCA was granted for this study.

#### 2.4.4. Subjective Cognitive and Mental Score (SS)

The SS was used to examine changes in the severity of psychiatric symptoms. The SS specifies a list of psychiatric and other symptoms subjectively perceived by the individual before the intervention and assesses the degree of change through surveys in cognitive and mental domains. Scores range up to 10, with lower scores indicating improvement in cognitive and mental domains.

#### 2.4.5. Canadian Occupational Performance Measure (COPM)

The COPM was used to examine changes in performance. This tool was developed in Canada and identifies problems in self-care, productive activities, and leisure activities through self-assessment [45]. Participants rank activities they perceive as problematic in the areas of self-care, leisure, and productivity and rate performance and satisfaction on a 10-point scale. Test–retest reliability ranges from 0.84 to 0.92, while internal consistency ranges from 0.56 to 0.71. In terms of score interpretation, higher performance and satisfaction indicate better outcomes. In this study, a high priority activity was selected and performance and satisfaction were compared before and after the intervention. Furthermore, a change score of two or more is considered clinically significant [46].

#### 2.4.6. Occupational Balance Questionnaire-Korean (OBQ-K)

The OBQ-K was used to examine changes in satisfaction with occupational balance. This tool was developed in 2014 to measure the overall satisfaction with occupational balance between individuals with autoimmune diseases and healthy individuals [47]. In this study, Park used the Korean version of the occupational balance questionnaire adapted for the Korean population (OBQ-K). The measure consists of a total of 13 self-report items, with each item scored from a minimum of 1 point (*strongly disagree*) to a maximum of 6 points (*strongly agree*). The test–retest reliability of the OBQ-K is 0.88, and score interpretation indicates that the total score ranges from 78 points, with higher scores reflecting higher levels of occupational balance [48].

### 2.5. Data Analysis

During the 22 sessions of motor and cognitive function, changes were analyzed with a graph that included the mean of each segment, two standard deviations (2SDs), and a trend line. Statistically significant changes were determined using the 2SD band method [49, 50]. A significant change in performance is indicated if at least two consecutive data points after the baseline phase fall outside the 2SD range. Changes in psychiatric symptoms, task performance, and occupational balance satisfaction were compared by analyzing the results of the pre- and postintervention assessments.

## 3. Result

### 3.1. Changes in Motor and Cognitive Function (22 Sessions)

#### 3.1.1. TUG

**Figure 2:**
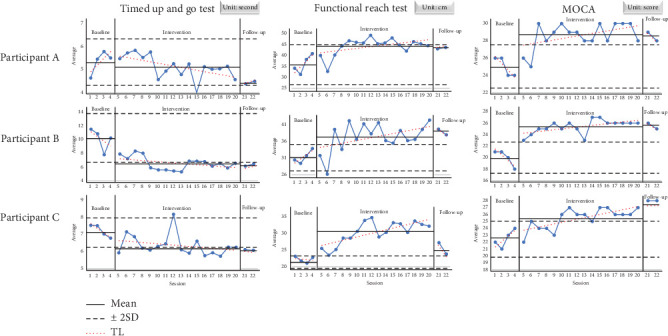
Changes in motor and cognitive function.

All subjects showed a decrease in mean execution time during the intervention period (mean 6.0 s) compared to the baseline period (mean 7.5 s) and a downward trend during the intervention period. Furthermore, this trend was maintained during the follow-up period (mean 5.6 s). Although Subject A showed a decreasing trend in execution time, it was not statistically significant (mean for Subject A across segments: 5.3, 5.1, and 4.4 s). However, Subjects B and C showed statistically significant results based on results within 2SDs or less in the intervention period compared to the baseline period using the 2SD band method. The average times for each segment were 10.1, 6.6, and 6.3 s for Subject B and 7.2, 6.4, and 6.1 s for Subject C, respectively (Figure 2).

#### 3.1.2. FRT

All subjects showed an increase in mean performance time during the intervention period (mean 37.0 cm) compared to the baseline period (mean 29.8 cm), with a rising trend line during the intervention period. This trend continued during the follow-up period (mean 35.7 cm). In addition, all subjects showed statistically significant results using the 2SD band method. The mean values for each segment were 36.1, 44.2, and 43.3 cm for Subject A; 31.2, 37.0, and 39.0 cm for Subject B; and 22.1, 30.0, and 25.3 cm for Subject C, respectively (Figure 2).

#### 3.1.3. Korean Version of MoCA

All subjects showed an increase in mean scores and trend lines during the intervention period (mean 26.5 score) compared to the baseline period (mean 22.5 score). Furthermore, this trend continued during the follow-up period (mean 27.3 score). In addition, all subjects showed statistically significant results according to the 2SD band method. The mean scores for each subject in each interval were as follows: Subject A showed scores of 25, 28.6, and 28.5; Subject B showed scores of 20, 25.4, and 25.5; and Subject C showed scores of 22.5, 25.4, and 28.0 (see Figure 2).

### 3.2. Comparison of Changes in Mental Symptoms, Task Performance, and Occupational Balance Before and After Intervention

#### 3.2.1. SS

All participants reported a subjective decrease in symptoms in both cognitive and mental domains. Participant A showed the greatest decrease (−3) in cognitive symptoms, while Participants B and C showed a similar decrease (−2). In terms of mental symptoms, Participants A and B showed the greatest decrease (−6), while Participant C showed a decrease of 1 point (Table 4).

#### 3.2.2. COPM

All subjects complained of difficulty in tasks related to walking (Table 1); all showed improvement in performance, with satisfaction only improving in two individuals. The performance improvement was most significant in Subjects A and B, showing the highest increase (+4), while Subject C showed an improvement of 2 points. The satisfaction improvement was most significant in Subject B, showing an increase of +4, followed by Subject A with an improvement of 2 points (Table 4).

#### 3.2.3. OBQ-K

All subjects showed an improvement in occupational balance satisfaction. Subject C showed the most significant change, increasing from 41 to 65, a difference of 24 points. Following this, Subject A showed an improvement of 8 points, increasing from 53 to 61, and lastly, Subject B showed a change of 6 points, decreasing from 43 to 28 (Table 4).

## 4. Discussion

Studies investigating the effects of BIG on various diseases are abundant, but there is a lack of research targeting patients with schizophrenia. Therefore, this study is aimed at examining the impact of BIG on the motor and cognitive enhancement of chronic schizophrenia patients on their mental symptoms, task performance, and occupational balance satisfaction. To achieve this, a single-subject experimental study was conducted on three subjects.

First, the TUG and the FRT were used to assess motor function. The TUG, which assesses dynamic balance and gait function, showed significant improvement in two subjects, and the FRT, which assesses dynamic balance, showed significant improvement in all subjects. In addition, the significant increase in dynamic balance in all subjects shown on the FRT is believed to have a significant impact on the improvement in gait function on the TUG (two subjects). This is thought to be due to the action of BIG, which requires many large muscles and balance maintenance in the standing position, resulting in improved postural stability, balance, and gait function. A study reporting improvements in FRT and functional gait assessment (FGA) scores by applying BIG to PD patients [31], as well as studies reporting improvements in TUG scores [31, 51, 52], reported similar results. In addition, a study using BIG in stroke patients reported improvements in Berg Balance Scale (BBS), TUG, and FRT in some subjects [53]. Thus, BIG was found to be effective not only in improving walking and balance in PD and stroke patients but also in schizophrenic patients.

The Korean version of the MoCA was then administered to determine changes in cognitive function. All subjects showed statistically significant improvement, and the rapid change from baseline to intervention is encouraging, although Subjects A and B tended to show a sharp decline in the baseline period. This is similar to previous studies using exercise therapies, including aerobic exercise, in patients with schizophrenia that have reported improvements in cognitive function [22–25]. This is likely due to the fact that BIG is characterized by loud singing during the intervention, and the breathing required to produce this vocalization is characteristic of aerobic exercise. In addition, previous studies have suggested that the intensity of aerobic exercise should be increased beyond VO_2_ max [22, 24, 25]. This suggests that the progression of BIG to higher intensities may have had a greater impact on cognitive function.

Finally, we examined changes in cognitive and mental symptoms, task performance, and satisfaction with occupational balance after mediation. First, all subjects showed a decrease in symptoms on the SS, which is used to identify mental health problems. In particular, two subjects showed a significant decrease in mental symptoms from a score of 7 (out of a maximum of 10) to 1. This indicates a significant improvement from a severe level to a mild level. This is consistent with previous studies showing that BIG is effective in reducing anxiety and depression in Parkinson's patients [54]. Cognitive problems also showed improvement, although the improvement in symptoms was relatively small compared to mental problems. However, this may be because all subjects had moderate levels of problems before the intervention, indicating a low possibility of change. Second, we identified each subject's important activities before the intervention and examined changes in task performance using the COPM. All subjects showed improvement in both performance and satisfaction scores. This is similar to a study that reported improvement in task performance in a BIG study of Parkinson's patients [53]. Subject C did not show any improvement in satisfaction after the intervention, despite already giving a satisfaction score of 10 out of 10, even though the performance score before the intervention was 8. However, the fact that all other subjects showed improvements of 2 points or more on the remaining items can be considered a statistically significant change [46]. This confirmed a meaningful change in performance. Third, satisfaction with occupational balance was assessed using the OBQ-K. All participants showed an improvement in their scores, with Participant C showing a significant improvement of 24 points. Participants A (preintervention score of 53) and B (preintervention score of 41) showed scores of 61 and 65, respectively, more than two-thirds of the maximum score of 78, indicating a positive change in their occupational balance. These results can be confirmed by a study applying BIG to Parkinson's patients, which reported improvements in daily life and quality of life through improved motor function [54]. Moreover, it is believed that the improvement in these symptoms will improve patients' psychological problems and affect their task performance, with confirmed improvements in motor function with TUG and FRT and cognitive function with MoCA. Furthermore, it is believed that this positive effect extends to their satisfaction with their occupational balance. This may be supported by a study reporting that regular exercise of appropriate intensity improves cognitive function such as memory and concentration, provides emotional stability, and positively affects mood, thus improving overall emotional well-being [40].

However, there are several limitations to this study as follows: (1) limited number of subjects making generalization difficult; (2) inability to explain the specific mechanisms of how motor and cognitive function improvements affect SS, COPM, and OBQ-K and which components of BIG have a greater impact; (3) lack of objective assessment using SS and detailed analysis of positive and negative symptoms; (4) failure to conduct a more analytical design through multiple baseline designs; (5) failure to include upper limb motor function; (6) lack of protocolized progression using recorded training videos for Home Work; (7) no mention of dopamine medication concentrations and no use of the Movement Disorder Society–Unified Parkinson's Disease Rating Scale (MDS-UPDRS) or Hoehn and Yahr (H&Y) scale to identify parkinsonism; and (8) lack of detail in the cognitive domain. Future research will need to take these limitations into account.

Nevertheless, this is the first study to use BIG in patients with chronic schizophrenia and to examine cognitive function in addition to motor function and how this affects psychiatric symptoms, task performance, and occupational balance. It is considered to be very meaningful. It is hoped that this will serve as a basis for further research in patients with schizophrenia and various psychiatric symptoms.

## 5. Conclusion

Schizophrenia is a psychiatric disorder with a wide range of symptoms. Primary drug therapy is more effective for positive symptoms than for negative symptoms, but it can also cause side effects. Therefore, the need for nonpharmacological interventions to improve symptoms, including negative symptoms, has increased. Recently, the importance of using BIG to reduce similar symptoms in Parkinson's patients and its potential for treating schizophrenia has been highlighted. In this study, BIG was found to promote improvement in motor and cognitive function in chronic schizophrenia patients, with positive effects on psychiatric symptoms, task performance, and occupational balance satisfaction. As a result, the potential of BIG as an intervention tool for schizophrenia patients was confirmed.

## Figures and Tables

**Table 1:** General characteristics of participant.

**Characteristics**	**Participant A**	**Participant B**	**Participant C**
Gender/age	Male/53	Male/27	Male/62
Marital status	Single	Single	Single
Education	University	High school	Elementary school
Onset (yr)	2008	2013	1986
Psychiatric symptoms	Anxiety, obsessions, poor impulse control	Anxiety, hallucinations, poor impulse control	Hallucinations, lethargy
Other symptoms	Decreased basic fitness, such as difficulty walking long distances, poor problem-solving skills, decreased ability to perform daily activities	Difficulty walking due to a narrow stride length, poor cognitive function, rheumatoid arthritis, decreased ability to perform daily activities	Decreased basic fitness, such as difficulty walking long distances, poor cognitive function, hypertension, diabetes mellitus, decreased ability to perform daily activities
Job	Part time (4 h/day)	Part time (4 h/wk)	Part time (4 h/wk)
COPM goal	Effective walking with direction apps	Avoid being late for work with a quick step	Walking safely and quickly

Abbreviations: COPM: Canadian Occupational Performance Measure, hr: hour, wk: week, yr: year.

**Table 2:** Components of LSVT-BIG program.

**Component**	**Description**
Maximal daily exercises	1. Floor to ceiling (8 repetitions)—seated position
	2. Side to side (8 repetitions)—seated position (repeat each left and right from here)
	3. Forward step and reach (8 repetitions)—standing position
	4. Sideways step and reach (8 repetitions)—standing position
	5. Backward step and reach (8 repetitions)—standing position
	6. Forward rock and reach (10 repetitions)—standing position
	7. Sideways rock and reach (10 repetitions)—standing position

Functional component tasks	Five functional component tasks

Hierarchy tasks	One to three hierarchy tasks are selected and are tailored to each individual

BIG walking	Distance/time may vary

**Table 3:** Program and hierarchy task components per participant.

	**Functional component tasks**	**Hierarchy tasks**
Participant A	Walking forward heel to toe on a line	Work with documents in Word
	Send and receive gym balls	
	Catch ball	
	Paper cutting with scissors	
	Practice typing	

Participant B	Walking forward heel to toe on a line	Meal preparation
	Send and receive gym balls	
	Paper cutting with scissors	
	Practice typing	
	Food ingredient shredding	

Participant C	Send and receive gym balls	Playing badminton
	Catch ball	
	Food ingredient shredding	
	Bouncing the shuttlecock	
	Jumping jack	

**Table 4:** Amount of change before and after intervention.

**Test classification**	**Participants**	**Preintervention**	**Postintervention**	**Amount of change**
SS (cognitive/mental score)	A	4/7	1/1	−3/−6
	B	5/7	3/1	−2/−6
	C	4/4	2/3	−2/−1

COPM (performance/satisfaction score)	A	3/5	7/7	+4/+2
	B	4/4	8/8	+4/+4
	C	8/10	10/10	+2/0

OBQ-K (total score)	A	53	61	+8
	B	43	48	+6
	C	41	65	+24

Abbreviations: COPM: Canadian Occupational Performance Measure, OBQ-K: Occupational Balance Questionnaire; SS: subjective cognitive and mental score.

## Data Availability

The data that support the findings of this study are available upon reasonable request but are not publicly accessible due to privacy or confidentiality concerns.
